# Supplementary material for: Helical versus Flat Bis-Ferrocenyl End-Capped Peptides: The Influence of the Molecular Skeleton on Redox Properties
Source: Molecules. 2022 Sep 19;27(18):6128. doi: 10.3390/molecules27186128 (PMC9503075; doi:10.3390/molecules27186128)
Supplement: Supplementary file 1 [file molecules-27-06128-s001.zip › molecules-1899810-supplementary.pdf]

Helical versus flat bis-ferrocenyl end-capped peptides: the influence of the molecular skeleton on the redox properties

Saverio Santi, [1,*] Barbara Biondi, [2] Roberta Cardena, [1] Annalisa Bisello, [1] Renato Schiesari, [1] Silvia Tomelleri, [1] Marco Crisma, [2] Fernando Formaggio [1,2]

[1] Department of Chemical Sciences, University of Padova, via Marzolo 1, 35131 Padova, Italy
[2] Institute of Biomolecular Chemistry, Padova Unit, CNR via Marzolo 1, 35131 Padova, Italy;
* saverio.santi@unipd.it

Characterization of Fc-CO-[(L-(Dap)]$_n$-NH-Fc (*n* = 1-4)

Fc-CO-[L-Dap(Boc)]-NH-Fc

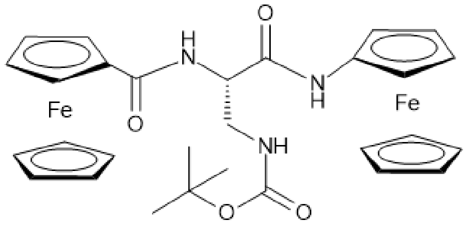

IR ($CDCl_3$, 1 mM) 3456, 3413, 3293, 1711, 1693, 1630, 1506 cm^{-1}. ^{1}H NMR ($CDCl_3$, 400 MHz): δ 8.71 (s, 1H, NH Fc), δ 7.75 (b, 1H, NH Dap), δ 5.48 (t, 1H, NH Boc), δ 4.83 (m, 2H, Fc), δ 4.69 (s, 2H, Fc), δ 4.54 (m, 1H, CH Dap), δ 4.41 (t, 2H, Fc), δ 4.25 (s, 5H, Fc), δ 4.18 (s,5H, Fc), δ 4.02 (m, 2H, Fc), δ 3.65 (m, 2H, CH_2 Dap), δ 1.50 (s, 9H, CH_3 Boc). HRMS (ESI+): m/z calcd for $C_{29}H_{33}N_3O_4Fe_2$, 599.12; found, 599.24.

Fc-CO-[L-Dap(Boc)]$_2$-NH-Fc

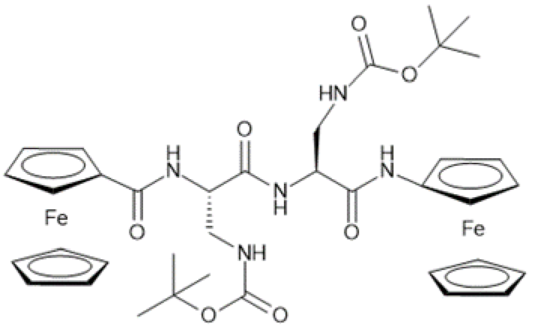

IR ($CDCl_3$, 1 mM) 3455, 3302, 1699, 1678, 1665, 1634, 1506 cm^{-1}. NMR ^{1}H ($CDCl_3$, 400 MHz): δ 8.53 (s, 1H, NH Fc), δ 8.40 (d, 1H, NH Dap_2), δ 8.24 (s, 1H, NH Dap_1), δ 5.20 (m, 1H, NH Boc Dap_1), δ 5.04 (m, 1H, NH Boc Dap_2), δ 4.91 (m, 2H, Fc), δ 4.83 (m, 2H, Fc), δ 4.59 (m, 1H, CH Dap_2), δ 4.39 (t, 2H Fc), δ 4.35 (m, 1H, CH Dap_1), δ 4.26 (s, 5H, Fc), 4.18 (s, 5H, Fc), δ 4.00 (b, 2H, Fc), δ 3.68 (m, 2H, CH_2 Dap_1), δ 3.62 (m, 1H, CH_2 Dap_2), δ 3.44 (m, 1H, CH_2 Dap_2), δ 1.54 (s, 18H, CH_3 Boc Dap_1), δ 1.54 (s, 18H, CH_3 Boc Dap_2). HRMS (ESI+): m/z calcd for $C_{37}H_{47}N_5O_6Fe_2$, 786.50; found, 786.17.

Fc-CO-[L-Dap(Boc)]$_3$-NH-Fc

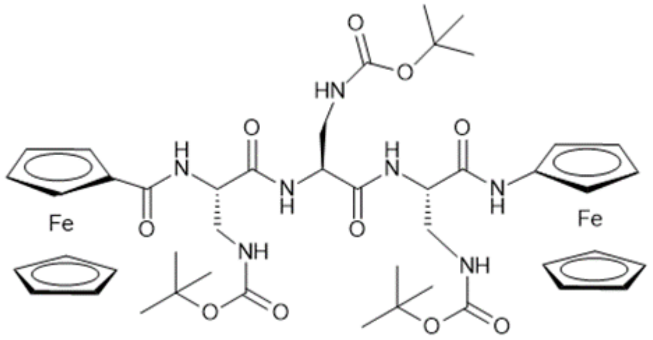

IR ($CDCl_3$, 1 mM) 3454, 3350, 3302, 1699, 1678, 1665, 1634, 1506 cm^{-1}. NMR ^1H ($CDCl_3$, 400 MHz): δ 8.75 (b, 1H, NH Dap$_3$), δ 8.42 (b, 1H, NH Dap$_2$), δ 8.00 (b, 1H, NH Dap$_1$), δ 7.99 (b, 1H, NH Fc), δ 6.08 (b, 1H, NH Boc Dap$_1$), δ 5.29 (b, 1H, NH Boc Dap$_2$), δ 5.06 (b, 1H, NH Boc Dap$_3$), δ 4.90 (s, 1H, Fc), δ 4.83 (b, 2H, Fc), δ 4.80 (b, 1H, Fc), δ 4.65 (m, 1H, CH Dap$_1$), δ 4.45 (m, 1H, CH Dap$_3$), δ 4.40 (b, 1H, Fc), δ 4.38 (m, 1H, Fc), δ 4.32 (b, 1H, CH Dap$_2$), δ 4.22 (s, 5H, Fc), δ 4.19 (s, 5H, Fc), δ 3.95 (b, 2H, Fc), δ 3.78 (m, 1H, CH_2 Dap$_1$), δ 3.68 (m, 2H, CH_2 Dap$_2$), δ 3.60 (m, 1H, CH_2 Dap$_3$), δ 3.53 (m, 1H, CH_2 Dap$_3$), δ 3.50 (m, 1H, CH_2 Dap$_1$), δ 1.54 (b, 9H, CH_3 Boc Dap$_2$), δ 1.47 (b, 9H, CH_3 Boc Dap$_1$), δ 1.20 (b, 9H, CH_3 Boc Dap$_3$). HRMS (ESI+): m/z calcd for $C_{45}H_{61}N_7O_{10}Fe_2$, 972.71; found, 972.32.

Fc-CO-[L-Dap(Boc)]$_4$-NH-Fc

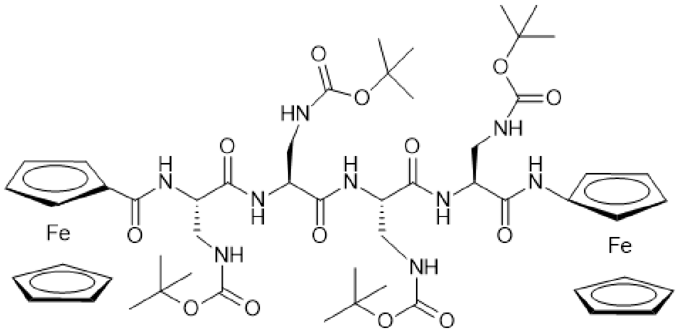

IR ($CDCl_3$, 1 mM) 3454, 3357, 3302, 1699, 1685, 1665, 1632, 1506 cm^{-1}. ^1H NMR (DMSO-d_6, 400 MHz): δ 9.16 (s, 1H, NH Fc), δ 8.11 (d, 1H, NH Dap$_2$), δ 7.99 (d, 1H, NH Dap$_3$), δ 7.94 (d, 1H, NH Dap$_4$), δ 7.76 (d, 1H, NH Dap$_1$), δ 6.95 (t, 1H, NH Boc Dap$_1$), δ 6.78 (t, 1H, NH Boc Dap$_2$), δ 6.70 (t, 1H, NH Boc Dap$_3$), δ 6.65 (t, 1H, NH Boc Dap$_4$), δ 4.81 (b, 1H, Fc), δ 4.79 (b, 1H, Fc), δ 4.67 (b, 1H, Fc), δ 4.58 (b, 1H, Fc), δ 4.40 (t, 2H, Fc), δ 4.36 (m, 1H, CH Dap$_1$), δ 4.32 (m, 1H, CH Dap$_4$), δ 4.29 (m, 1H, CH Dap$_2$), δ 4.27 (m, 1H, CH Dap$_3$), δ 4.22 (s, 5H, Fc), δ 4.10 (s, 5H, Fc), δ 3.94 (t, 2H, Fc), δ 3.43 (m, 1H, CH_2 Dap$_1$), δ 3.42 (m, 1H, CH_2 Dap$_2$), δ 3.40 (m, 1H, CH_2 Dap$_3$), δ 3.39 (m, 1H, CH_2 Dap$_4$), δ 3.35 (m, 1H, CH_2 Dap$_1$), δ 3.30 (m, 1H, CH_2 Dap$_2$), δ 3.26 (m, 1H, CH_2 Dap$_4$), δ 3.22 (m, 1H, CH_2 Dap$_3$), δ 1.38 (m, 9H, CH_3 Boc), δ 1.36 (m, 27H, CH_3 Boc). HRMS (ESI+): m/z calcd for $C_{53}H_{75}N_9O_{13}Fe_2$, 1157.74; found, 1158.42.

Table S1. Crystal data and structure refinement for **2**.

Identification code	mc308	
Empirical formula	$C_{37}H_{47}Fe_2N_5O_7 \times CDCl_3$	
Formula weight	905.87	
Temperature	293(2) K	
Wavelength	0.71073 Å	
Crystal system	Monoclinic	
Space group	P 21	
Unit cell dimensions	a = 10.7188(4) Å	a= 90°.
	b = 13.0060(3) Å	b= 97.356(3)°.
	c = 15.9072(6) Å	g = 90°.
Volume	2199.35(13) $Å^3$	
Z	2	
Density (calculated)	1.368 Mg/m^3	
Absorption coefficient	0.892 mm^{-1}	
F(000)	940	
Crystal size	0.35 × 0.10 × 0.04 mm^3	
Theta range for data collection	2.474 to 29.252°.	
Index ranges	$-14 \leq h \leq 14$, $-16 \leq k \leq 17$, $-21 \leq l \leq 21$	
Reflections collected	38968	
Independent reflections	10633 [R(int) = 0.0447]	
Completeness to theta = 25.242°	99.9 %	
Absorption correction	Semi-empirical from equivalents	
Max. and min. transmission	1.00000 and 0.81394	
Refinement method	Full-matrix least-squares on F^2	
Data / restraints / parameters	10633 / 133 / 487	
Goodness-of-fit on F^2	1.023	
Final R indices [I>2sigma(I)]	R_1 = 0.0504, wR_2 = 0.1197	
R indices (all data)	R_1 = 0.0819, wR_2 = 0.1383	
Absolute structure parameter	-0.027(7)	
Extinction coefficient	n/a	
Largest diff. peak and hole	0.437 and -0.353 e.$Å^{-3}$	

Table S2. Selected backbone and side-chain torsion angles [°] for **2**.

C01-C0-N1-C1A	ω_0	-172.0(4)
C0-N1-C1A-C1	ϕ_1	-79.4(5)
N1-C1A-C1B-N1G	χ_1^1	67.0(5)
C1A-C1B-N1G-C101	χ_1^2	-86.7(5)
C1B-N1G-C101-O102	χ_1^3	-167.8(4)
N1-C1A-C1-N2	ψ_1	-2.8(6)
C1A-C1-N2-C2A	ω_1	173.0(4)
C1-N2-C2A-C2	ϕ_2	-79.9(5)
N2-C2A-C2B-N2G	χ_2^1	83.4(5)
C2A-C2B-N2G-C201	χ_2^2	-70.8(6)
C2B-N2G-C201-O202	χ_2^3	-178.2(4)
N2-C2A-C2-NT	ψ_2	-10.1(5)
C2A-C2-NT-CT1	ω_T	174.1(4)

Table S3. Hydrogen bonds for **2** [Å and °].

D-H...A	d(D-H)	d(H...A)	d(D...A)	<(DHA)
NT-HT...O0	0.86	2.47	3.304(5)	163.3
N1-H1...O101	0.86	2.11	2.859(5)	145.0
N2-H2...O201	0.86	2.07	2.797(5)	141.2
N1G-H1G...O2#1	0.86	2.20	2.981(5)	150.5
N2G-H2G...O1#2	0.86	2.02	2.816(5)	153.1
C1S-D1S...O0	0.98	2.05	3.023(7)	175.6

Symmetry transformations used to generate equivalent atoms:

#1 -x+2,y+1/2,-z+1 #2 -x+2,y-1/2,-z+1

Packing. In the packing mode, intermolecular H-bonds are observed between the side-chain NH groups of Dap(1) and Dap(2) as the donors, and the (backbone) O2 and O1 carbonyl oxygen atoms, respectively, as the acceptors (Table S3), connecting molecules related through the two-fold screw axis along the b direction.

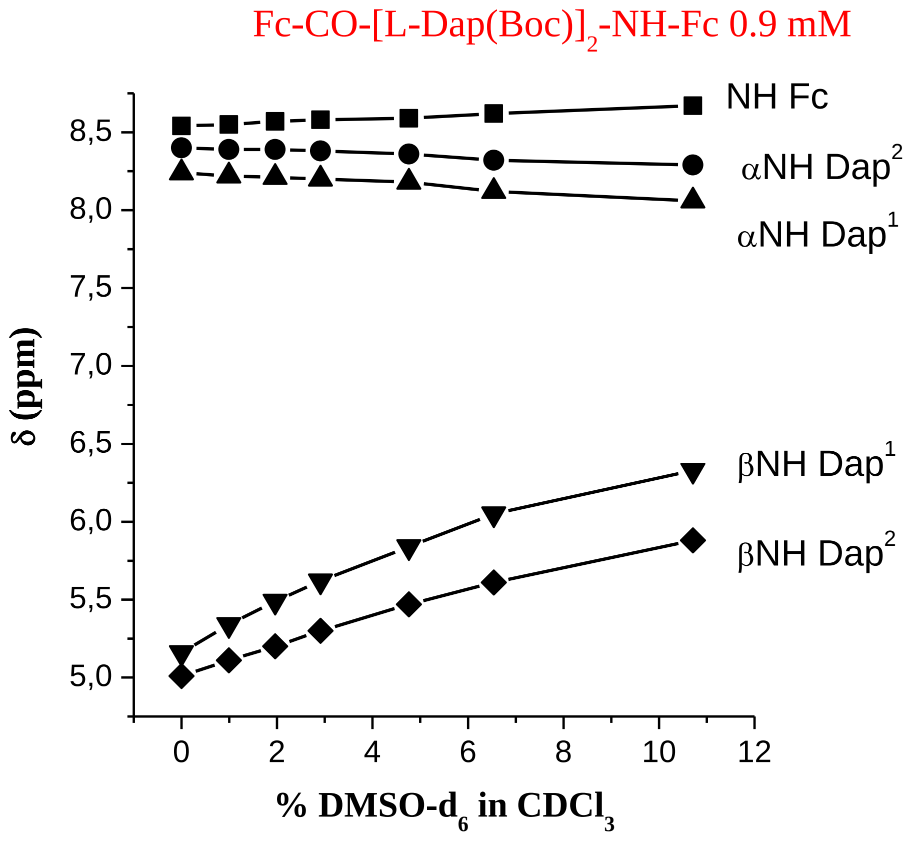

Figure S1. Variation of the amide and urethane NH chemical shifts upon addiction of DMSO to a solution of peptide **2** in $CDCl_3$.

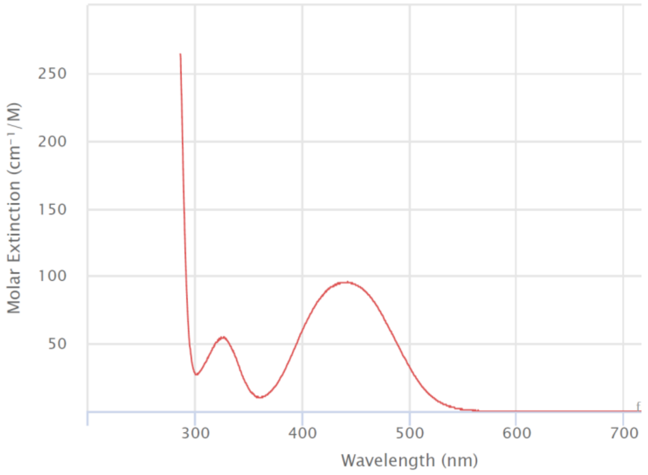

Figure S2. Absorption spectrum of ferrocene in the 300-600 nm region in cyclohexane solution (https://omlc.org/spectra/PhotochemCAD/html/062.html).

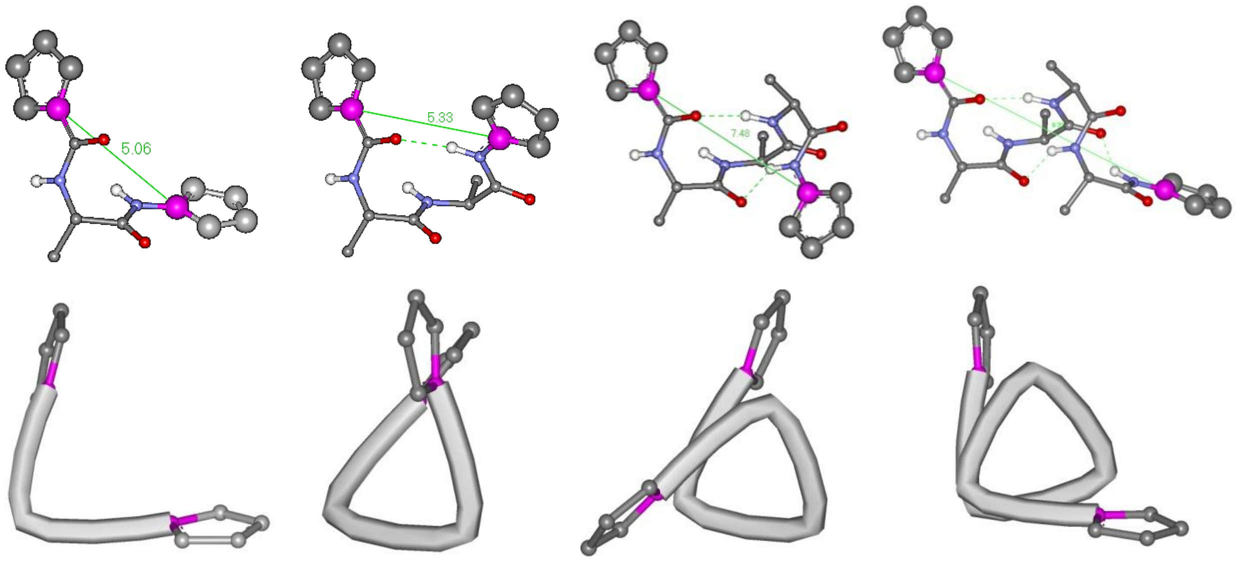

Figure S3. 3_{10}-Helical models for **1–4** (left to right); for clarity, only one Fc ring is drawn. Top row: side view of the four compounds; in **2** and **4** the two Fc units are directly connected via one and two H-bonds, respectively. Bottom row: views along the helix axis; in **1** and **4** the aromatic rings of Fc are roughly perpendicular, in **2** and **3** they are almost parallel. WebLab ViewerPro 3.7 software (Molecular Simulations Inc.)
